# Drain Versus No Drain in Open Mesh Repair for Incisional Hernia, Results of a Prospective Randomized Controlled Trial

**DOI:** 10.1007/s00268-022-06725-4

**Published:** 2022-12-15

**Authors:** Mélissa Willemin, Clara Schaffer, Amaniel Kefleyesus, Anna Dayer, Nicolas Demartines, Markus Schäfer, Pierre Allemann

**Affiliations:** 1 Department of Visceral Surgery, Lausanne University Hospital CHUV, Rue du Bugnon 46, 1011 Lausanne, Switzerland; 2 Department of Surgery, Riviera-Chablais Hospital, Rennaz, Switzerland; 3 Department of Surgery, Clinique La Source, Lausanne, Switzerland

## Abstract

**Background:**

Open mesh repair of incisional hernia is associated with different local complications, particularly bleeding and seroma formation. Traditionally, drains have been placed perioperatively to prevent these complications, despite the lack of scientific evidence or expert consensus. We formulated the hypothesis that the absence of drainage would reduce number of patients presenting collections or complications. The present study aimed to compare postoperative complication rates after open mesh repair for incisional hernia with or without prophylactic wound drainage.

**Methods:**

Prospective randomized study using standardized surgical technique and drain placement. The primary endpoint was the evaluation of residual fluid collection with ultrasound on postoperative day 30. Other complications, subdivided into medical and surgical, were analyzed as secondary endpoints.

**Results:**

There were 144 patients randomized (70 with drain, 74 without drain). No difference was identified between both groups for fluid collection at 30 days (60.3% vs. 62%, *p* = 0.844). However, less surgical complications were identified in the drain group (21.7% vs. 42.7%, *p* = 0.007), with a lower wound dehiscence rate (1.5% vs. 9.3%, *p* = 0.041).

**Conclusions:**

Prophylactic drainage in open incisional hernia repair does not objectively reduce the rate of postoperative fluid collections. Therefore, our results do not support the use of routine drainage in incisional hernia repair.

**Trial registration:**

Trial registration on clinicaltrials.gov (NCT00478348).

**Supplementary Information:**

The online version contains supplementary material available at 10.1007/s00268-022-06725-4.

## Introduction

Incisional hernia formation presents a major burden of care after open abdominal surgery. It may occur in up to 20% of patients after median laparotomy [1–4] and the risk factors have been well described [3, 5]. It is estimated that approximately 100,000 incisional hernia repairs are performed annually in the United States [2, 3, 6]. In Europe, approximately 3900 incisional hernia repairs are performed each year in Netherland and 1800 were performed in Denmark between 2007 and 2008 [7, 8].

The most common complications after open mesh incisional hernia repairs are surgical site infections and collections [4, 9]. Drains are commonly used to decrease the occurrence of postoperative fluid collections in the surgical field even though the exact etiology of seroma formation remains controversial. Klink et al. reported that the nature of the fluid collection could predict the subsequent development of a seroma [10]. Whether or not drains have an influence on the formation of fluid collections is an ongoing debate and experts have not yet reached a consensus. Some authors suggest that wound infections are more frequent and that complications are not reduced with the use of drains [11], while others argue that even if drains don’t prevent infections, they may be beneficial [12]. In 2017, the Australian Supreme Court debated this subject and ruled that standard management requires the placement of a drain in complex cases [13]. A survey among members of the Australian Society of General Surgery regarding the placement of drains in incisional hernia repair outlined a variety of practices and beliefs [14]. This result was confirmed by a retrospective audit which concluded that drains were placed in only a minor proportion of cases [15].

Indeed there are few prospective studies comparing outcomes with and without drains after incisional hernia repair [16, 17]. In 2015, a Brazilian group prospectively compared drains and quilting sutures in incisional hernia repair and found similar rates of seroma and surgical wound infections [18].

We formulated the hypothesis that the absence of drainage would not influence the number of patients presenting collections or complications after incisional hernia repair. This current prospective, randomized controlled trial aimed to determine the influence of prophylactic drainage in incisional hernia repair on postoperative fluid collections and complications.

## Material and methods

### Compliance with ethical standards

This prospective randomized multicentric study was conducted according to the CONSORT statement guidelines [19]. The study was approved by the local ethic committee (CER-VD 31/07, DP-2007-CHV-UNIL) and was registered as a prospective randomized clinical trial on clinicaltrials.gov (NCT00478348).

### Patients

Patients were screened preoperatively at the outpatient clinic of the Department of visceral surgery, Lausanne University Hospital CHUV. Inclusion criteria were patients between 20 and 80 years old requiring an elective open incisional hernia repair with a physical status classification according to the American Society of Anesthesiologists (ASA) between 1 and 3. Exclusion criteria were a hernia defect of less than 2 cm, inguinal hernia, ongoing antibiotic treatment before admission, and emergency surgery for an incarcerated or strangulated hernia. In addition, we excluded laparoscopic hernia procedures and patients under immunosuppressive therapy.

All included patients completed an informed consent form validated by the local ethics committee.

### Sample size calculation

The complication rate in the drainage group was estimated to reach 20%. No prospective study comparing drainage and without drainage was available in the literature when the study was started. Rates of complications were estimated according to a consensus between surgeons participating to the study. We hypothesized that the absence of drainage would decrease the occurrence of either surgical site infection, seroma, hematoma, or early hernia recurrence by 50% within 30 days after surgery. The sample size was calculated with an 80% power; and a significance level with a *p*-value under 0.05 was established. The sample size calculation according to power yielded 398 patients. Approximately 60 patients per year present an incisional hernia requiring surgery. With a participation rate of 80% among eligible patients, the study was estimated to last over 8 years. A second hospital was included to reduce the study duration. The study was however prematurely interrupted due to the emergence of concurrent minimally invasive techniques and the number of 398 patients was not reached. Despite laparoscopic intraperitoneal onlay mesh (IPOM) technique has been known for more than 20 years, the penetration and adoption of this technique required some time to be achieved in our teaching department. However, laparoscopic IPOM techniques have become very popular during the study period and compete with our recruitment.

### Surgical techniques and randomization

Patients were electronically randomized on the day before surgery. They were allocated to the treatment group (with drainage) or the control group (without drainage). A clinical nurse performed the randomization through a web-based service (https://www.randomizer.at—Institute for Medical Informatics Statistics and Documentation, Medical University of Graz, Austria).

This study was not blinded due to technical limitations. Indeed, it would have been difficult to blind the operators placing a drain and to blind the patients due to the presence of dressings and drain bottles.

The surgical technique was standardized in both groups. All patients underwent an open repair according to the Rives-Stoppa procedure [20]. This procedure consists of the placement and fixation of a mesh in the retromuscular position after dissection and reduction of the hernial sac and closure of the posterior sheath. The closure of the anterior sheath with or without relaxing incisions allows for closure above the mesh. Mesh type choice was at the discretion of the surgeon. Redon drains (10 French, closed active-suction drainage) were placed in the retromuscular and preaponeurotic (subcutaneous) planes. In the drainage group, drains were removed sequentially when the output was <30 ml/day. This volume was defined by a consensus between surgeons participating to the study because no data exist in the literature about volume or duration of drainage.

### Endpoints

The endpoints were registered during a follow up period of thirty days. The primary endpoint was the presence of a fluid collection on postoperative day 30 (POD 30). All patients underwent an ultrasound to objectively assess the presence or absence of a collection. Collections were subdivided into seroma, hematoma and abscess. Seroma was defined as a subcutaneous fluid collection without a solid component. The hematoma was characterized by the presence of debris, and abscess was confirmed by the presence of pus on a puncture.

Secondary endpoints were complications, classified according to Clavien-Dindo classification [21] and Comprehensive Complication Index (CCI) [22]. Complications were pre-defined when the study was elaborated and more than one complication was registered per patient in order to calculate Clavien-Dindo score but also the CCI. These complications were also subdivided in a second time into surgical versus medical complications. Surgical complications included early recurrence, surgical site infection classified according to Center for Disease Control and Prevention (CDC) criteria [23], evisceration, wound dehiscence, or ileus. Medical complications such as cardiac arrhythmia or ischemia, pneumonia, pulmonary edema, thromboembolic events, urinary tract infections or obstructions, and withdrawal syndrome were also reported.

### Statistical analysis

The null hypothesis was the absence of difference between both groups and the alternative hypothesis was a decrease of complications without drain. The normal distribution of the variables was assessed with a Kolmogorov–Smirnov test. Continuous variables were analyzed with a Student’s t-test and categorial binary variables with a Pearson’s chi-square test or a Fisher’s exact test. A *p*-value smaller than 0.05 was considered statistically significant.

## Results

### Demographics

**Fig. 1:**
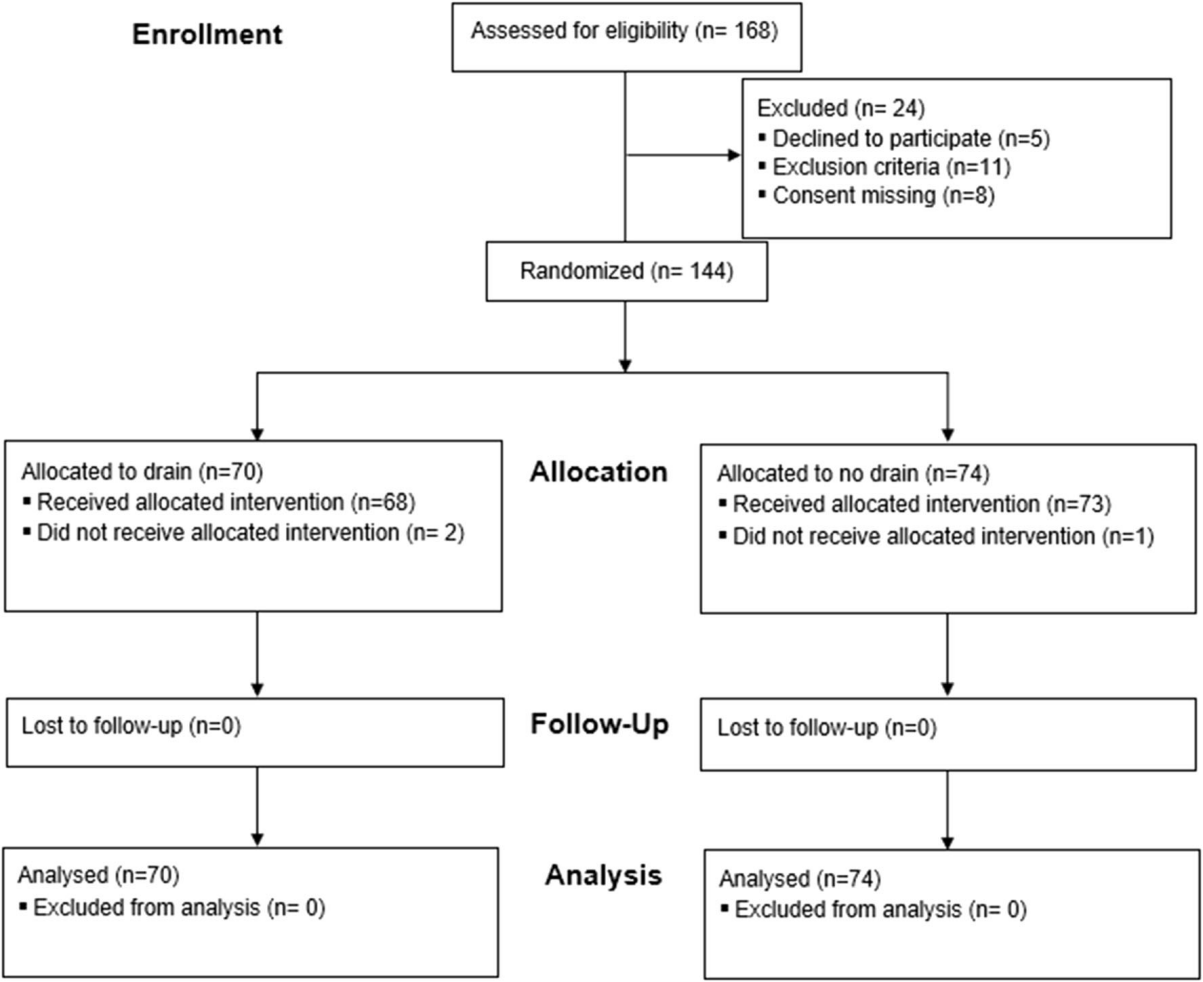
Flowchart of patient’s eligibility

From October 2007 to January 2017, 144 patients (80 males, 64 females) with a median age of 64 years were included according to the eligibility criteria (Fig. 1). Patient’s demographics are presented in Table 1. Both groups were comparable.

**Table 1:** Patients demographics

	Drain group (%) *N* = 70	No drain group (%) *N* = 74	*p*-value
Age, year (mean)	61.8	62.1	0.914
BMI, kg/m^2^ (mean)	28.4	29.8	0.626
Diabetes	7 (10.1)	13 (17.3)	0.213
COPD	17 (24.6)	11 (14.7)	0.131
Current smoker	30 (45.5)	24 (34.8)	0.206
CHF	0	3 (4)	0.096
ASA class			
1	2 (3.0)	3 (4.2)	0.721
2	39 (59.1)	46 (63.8)	0.563
3	24 (36.4)	23 (32.0)	0.584
Recurrent hernia	11 (15.9)	14 (18.7)	0.666
Hernia defect, cm (mean)	8.13	7.90	0.804
<5 cm	11 (20.8)	16 (28.6)	0.345
5–10 cm	21 (39.6)	23 (41.1)	0.878
>10 cm	21 (39.6)	17 (30.3)	0.310
Charlson comorbidity index (mean)	7.91	12.80	0.831

*BMI* body mass index, *COPD* chronic obstructive pulmonary disease, *CHF* cardiac heart failure with ejection fraction <35%, *ASA* physical status classification according to American Society of Anesthesiologists, *p*-value with level of significance at 0.05

### Endpoints

The rate of fluid collection at POD 30, which was defined as the primary endpoint, was high in both groups, but the difference between both groups (60.3% drain group vs. 62.0% no drain group, *p* = 0.844) was not statistically significant. The median drainage duration was 4 days. No difference was found when each subtype of complication was considered independently: seroma (46% drain group vs. 45.1% no drain group, *p* = 0.911), hematoma (11.1% drain group vs. 16.9% no drain group, *p* = 0.338) and abscess (4.8% drain group vs. 4.2% no drain group, *p* = 0.881). We recorded the size of all collections and performed analysis with different volume categories, without finding any difference between the groups. No difference in terms of collection was demonstrated based on hernia defect size. However, there was a statistically significant reduction in overall complications on POD 30 in the drain group (29% vs. 50.7%, *p* = 0.008), with a lower mean CCI (7.3% vs. 24%, *p* = 0.006) and a lower rate of surgical complications (21.7% vs. 42.7%, *p* = 0.007). Both groups were similar regarding medical complications as depicted in Table 2. Length of hospital stay and readmission rates were similar in both groups. The operative mean time was 151 and 135 min for the drain and no-drain group, respectively. Details of all complications are available in the supplementary material.

**Table 2:** Operative and postoperative characteristics

	Drain group (%) *N* = 70	No drain group (%) *N* = 74	*p*-value
Collections			
Collection	38 (60.3)	44 (62.0)	0.844
Hernia defect <5 cm	7 (70.0)	9 (60.0)	0.691
Hernia defect 5–10 cm	12 (63.2)	16 (69.6)	0.661
Hernia defect >10 cm	12 (60)	11 (64,7)	0.769
Seroma	29 (46.0)	32 (45.1)	0.911
Hematoma	7 (11.1)	12 (16.9)	0.338
Abscess	3 (4.8)	3 (4.2)	0.881
Complications			
Overall complications	20 (29.0)	38 (50.7)	0.008
Surgical complications	15 (21.7)	32 (42.7)	0.007
Wound dehiscence	1 (1.5)	7 (9.3)	0.041
Ileus	4 (6.0)	7 (9.5)	0.440
Early recurrence (<POD 30)	1 (1.6)	3 (4.1)	0.377
Medical complications	8 (11.6)	10 (13.3)	0.753
Antibiotic therapy	7 (10.1)	11 (14.7)	0.412
Surgical site infection	4 (5.9)	7 (9.3)	0.439
Superficial	3 (4.4)	6 (8.0)	0.378
Deep	1 (1.5)	4 (5.3)	0.209
Organ/space	1 (1.5)	0	0.292
Clavien-Dindo classification			
I	6 (8.7)	14 (18.7)	0.084
II	9 (12.9)	16 (21.6)	0.190
IIIa	0	0	
IIIb	6 (8.7)	13 (17.3)	0.126
IVa	2 (2.9)	0	0.138
IVb	2 (2.9)	1 (1.3)	0.503
V	0	0	
Comprehensive complication index			
0–25	61 (88.4)	56 (74.7)	0.035
25–45	5 (7.3)	18 (24)	0.006
45–100	3 (4.3)	1 (1.3)	0.271
Operative time (min)	151	135	0.073
Hospital stay (days)	7.23	7.18	0.970
Readmission	8 (11.9)	11 (15.5)	0.545

Surgical site infection according to Center for Disease Control and Prevention (CDC) criteria, *p*-value with level of significance at 0.05

### Follow up

The follow up was complete for all patients, with a clinical and radiological control at POD 30.

## Discussion

This prospective randomized study assessed the role of intraoperatively placed drains to avoid postoperative fluid collection after open incisional hernia repair. Postoperative fluid collections were observed in both groups in up to 65% of patients, and drain placement could not decrease the incidence of that particular complication at POD 30. This rate of collection seems important; however, only a small amount was clinically relevant. Although the trial was negative on the primary endpoint, the secondary endpoints highlighted significant results in terms of morbidity. There were significantly fewer overall complications on POD 30 in the drain group and a lower rate of surgical complications. These findings will be discussed in more detail below.

There is no clear evidence in the current literature regarding the prophylactic use of drains. In a prospective study including 42 patients in 2015, Westphalen et al. reported a similar rate of seroma in patients with drains (52.4%) versus quilting sutures (42.9%) on POD 30. A retrospective study of Krpata et al. in 2017 analyzed complications after drainage in incisional hernia repair with a retromuscular mesh [24]. This large study (*n* = 581) with 82.8% of drainage use showed a preventing role for seroma development. However, their rates of seroma in the drainage group (1%) and the control group (8%) were largely inferior to our findings, most likely related to diagnostic methods and definitions used by the authors to detect seroma. This difference calls into question the clinical relevance of fluid collection, which may be clinically silent while radiologically detectable. Moreover, we have to bear in mind the methodological difference in drain placement. The difference observed between the findings of Krpata et al. and ours could be also explained by the number and different sites of drainage between our studies. For example, the retromuscular drainage is a less extensible space than the subcutaneous, and therefore less prone to large collections.

The high complication rate in our study (29% in the drainage group; 50.7% in the control group) is explained by a compulsory and institutionally-organized system of complication recording, including minor complications graded I and II according to Clavien-Dindo classification. The higher rate of surgical complications in the group without drainage might suggest that drainage could prevent complications other than fluid collection, and therefore positively influence postoperative outcomes. Although drainage was predictive of a lower CCI, it did not reflect on hospital stay or readmission rate. In addition, the severity of complications graded by Clavien-Dindo classification was similar in both groups.

We investigated which subtype of complication was most present in each group. The surgical site infection rate was comparable in both groups (5.9% vs. 9.3%, *p* = 0.439), remarkably close to literature [25], especially to reports from Krpata et al. (6–3%) and Westphalen et al. (19.1–23.8%). Although the absence of drainage seems to be a predictive factor for wound dehiscence according to our results (*p* = 0.041), these dehiscences only required local care and were not associated with more surgical site infection. These results may assume that drains may prevent local fluid retention, while the absence of drainage may favor the evacuation of remaining fluid through the wound, translating into wound dehiscence, ultimately delaying wound healing. Although a statistically significant difference is found for surgical complications (*p* = 0.007), in our opinion, no strong recommendation can be made with our results given that the subcategories show only slightly significant differences (*p* = 0.041).

The strength of the present study relies on its high-standard prospective, randomized design and also on the recording of objective and quantifiable endpoints. External validity is high since inclusion criteria did not select the type of initial surgery or patient comorbidities. The sample represents a standard population making it easily reproducible for further investigation. The 10-year recruitment period is a drawback since laparoscopic surgeries have become widely popular. These minimally-invasive procedures competed with our study and limited recruitment. After careful evaluation of this situation with our ethical committee, the study had to be terminated prematurely and the number of participants estimated by the initial power calculation has not been reached. In our opinion, the value of the present study remains strong, as these novel approaches are still not widely accepted or implemented, and open surgery remains the gold standard for the majority of surgeons. In addition, the 144 included patients represent a large population when compared to other series. Since this study was terminated prematurely due to the expansion of minimally invasive techniques, it would be interesting to repeat this research with other techniques, such as minimal invasive approaches or onlay mesh placement. Finally, according to our primary endpoint, the study was negative. However, a clinically relevant conclusion is in our view appropriate.

## Conclusion

Prophylactic drainage in open incisional hernia repair does not objectively reduce the rate of postoperative fluid collections. Therefore, our results do not support the use of routine drainage in incisional hernia repair.

## Supplementary Information

Below is the link to the electronic supplementary material.

Supplementary file1 (DOCX 17 KB)
