# Supplementary material for: Drain Versus No Drain in Open Mesh Repair for Incisional Hernia, Results of a Prospective Randomized Controlled Trial
Source: World J Surg. 2022 Dec 15;47(2):461–8. doi: 10.1007/s00268-022-06725-4 (PMC9803733; doi:10.1007/s00268-022-06725-4)
Supplement: Supplementary file 1 — Supplementary file1 (DOCX 17 KB) [file 268_2022_6725_MOESM1_ESM.docx]

**Drain vs no drain in open mesh repair for incisional hernia, Results of a prospective randomized controlled trial**

World Journal of Surgery

Mélissa Willemin*,1, Clara Schaffer*,1, Amaniel Kefleyesus1, Anna Dayer2, Nicolas Demartines1, Markus Schäfer1, Pierre Allemann1,3

* Mélissa Willemin and Clara Schaffer have equal contribution to this work. 1 Department of Visceral Surgery, Lausanne University Hospital CHUV, Lausanne, Switzerland 2 Department of surgery, Riviera-Chablais Hospital, Rennaz, Switzerland 3 Department of surgery, Clinique La Source, Lausanne, Switzerland

*Correspondence to*: Nicolas Demartines, MD, FACS, FRCS, Professor and Chairman, Department of Visceral Surgery, Lausanne University Hospital CHUV, Rue du Bugnon 46, 1011 Lausanne, Switzerland, Phone: +41 21 314 24 00, Fax: +41 21 314 24 11, E-mail: [demartines@chuv.ch](mailto:demartines@chuv.ch)

| **Supplementary material** Report of all postoperative complications (n = 144) | | |
| --- | --- | --- |
| Clavien-Dindo classification | Description of event | Description of intervention |
| I | Wound dehiscence | Observation |
| I | Subcutaneous hematoma | Bedside drainage |
| II | Urinary retention | Bladder catheterization |
| IIIb, IVa | Abdominal wall necrosis due to surgical site infection, nosocomial pneumoniae | Surgery, intensive care unit transfer |
| IIIb | Abdominal wall abscess | Surgery |
| I | Subcutaneous seroma | Bedside drainage |
| II | Lower urinary tract infection | Antibiotic therapy |
| IIIb | Abdominal wall hematoma | Surgery |
| IIIb | Abdominal wall abscess | Surgery |
| II | Paralytic ileus | Nasogastric tube placement |
| I | Subcutaneous seroma | Bedside drainage |
| I | Subcutaneous seroma | Bedside drainage |
| I | Abdominal wall abscess | Bedside drainage |
| II, II | Pulmonary embolism, paralytic ileus | Medical treatment, nasogastric tube placement |
| IIIb | Abdominal wall hematoma | Surgery |
| I | Subcutaneous seroma | Bedside drainage |
| IIIb | Subcutaneous hematoma | Surgery |
| IVa | Atrial fibrillation with ventricular tachycardia response | Medical treatment |
| II | Superficial site infection | Antibiotic therapy |
| I | Sero-purulent discharge | Observation |
| I | Subcutaneous hematoma | Observation |
| I | Abdominal wall abscess | Bedside drainage |
| II, II | Paralytic ileus, abdominal wall abscess | Bedside drainage and antibiotic therapy, nasogastric tube placement |
| IIIb | Abdominal wall hematoma | Surgery |
| II, II | Alcoholic withdrawal, volume overload | Medical treatment |
| I | Wound dehiscence | Observation |
| II | Urinary retention | Bladder catheterization |
| II | Lower urinary tract infection | Antibiotic therapy |
| II | Paralytic ileus | Nasogastric tube placement |
| IIIb | Subcutaneous abscess | Surgery |
| I | Subcutaneous hematoma | Observation |

| IIIb | Subcutaneous hematoma | Surgery |
| --- | --- | --- |
| I | Subcutaneous abscess | Bedside drainage |
| I | Wound dehiscence | Wound care |
| I | Wound dehiscence | Wound care |
| II | Paralytic ileus | Nasogastric tube placement |
| II | Urinary retention | Bladder catheterization |
| II, IVb, IIIb | Urinary retention, acute coronary syndrome, septic shock due to surgical site infection | Bladder catheterization, coronarography, intensive care unit transfer |
| IIIb | Incisional hernia recurrence | Surgery |
| II | Urinary retention | Bladder catheterization |
| II, II | Surgical site bleeding, nosocomial pneumoniae | transfusion of one red blood cell concentrate, antibiotic therapy |
| II | Paralytic ileus | Nasogastric tube placement |
| II, II, II, IIIb, I | Urinary retention, lower urinary tract infection, paralytic ileus, incisional hernia recurrence, wound dehiscence | Bladder catheterization, antibiotic therapy, nasogastric tube placement, surgery |
| I | Abdominal wall hematoma | Observation |
| II | Paralytic ileus | Nasogastric tube placement |
| IIIb | Enterocutaneous fistula | Surgery |
| IIIb | Abdominal wall abscess | Surgery |
| II | Urinary retention | Bladder catheterization |
| IIIb, II | Subcutaneous hematoma, nosocomial pneumoniae | Surgery, antibiotic therapy |
| II, II | Urinary retention, nosocomial pneumoniae | Bladder catheterization |
| IIIb, I | Abdominal wall necrosis due to surgical site infection, wound dehiscence | Surgery |
| I | Wound dehiscence | Wound care |
| IIIb | Subcutaneous hematoma with surgical site infection | Surgery |
| II | COPD exacerbation | Antibiotic therapy |
| I | Wound dehiscence | Wound care |
| II | Paralytic ileus | Nasogastric tube placement |
| II, IIIb, IVb | Paralytic ileus, incisional hernia recurrence with jejunal incarceration, necrotizing pneumonia on bronchoaspiration | Nasogastric tube placement, surgery, surgery, intensive care unit transfer |
| II, IIIb | Paralytic ileus, incisional hernia recurrence | Nasogastric tube placement, surgery |
| IVb | Transverse colon perforation | Surgery and transfer intensive care unit |

COPD : chronic obstructive pulmonary disease
